# Hospitalization dynamics during COVID-19: Insights into disease trends and patient outcomes

**DOI:** 10.1371/journal.pone.0321269

**Published:** 2025-04-28

**Authors:** Mahsa Motiei, Afagh Hassanzadeh Rad, Hamidreza Badeli, Reza Bayat

**Affiliations:** Pediatric Diseases Research Center, Guilan University of Medical Sciences, Rasht, Iran; University of Tabuk, SAUDI ARABIA

## Abstract

**Objectives:**

To find the pattern of hospitalization pattern change in COVID-19 pandemic, we aimed to compare the admission and mortality rate of each disease in all wards before and during the pandemic.

**Methods:**

Data for all ICD-10 disease categories were collected from 17 shahrivar hospital database for 14922 patients before (23 July 2017–23 January 2020 (and 10941 patients during the pandemic (20 February 2020- 20 September 2022). We compared the age, sex, duration of hospitalization, the frequency of readmission and outcome of patients in these two periords. Also the number of patients in each ICD-10 category and in each ward was compared.

**Results:**

Comparing the two periods revealed a decrease in overall admission frequency (14,922 vs. 10,941 patients). During the pandemic, patients experienced significantly shorter hospital stays (P < 0.001). There was no significant difference in the number of patients entering remission or experiencing mortality (P = 0.063). Notably, admissions for neoplasms, blood disorders, nervous system conditions, eye disorders, circulatory and digestive system issues, genitourinary system disorders, congenital malformations, and poisoning significantly increased during the pandemic, while admissions for other conditions decreased. Admissions varied significantly across departments, with notable increases in the NICU, PICU, emergency, neonatal, and hematology departments during the pandemic (P < 0.001).

**Conclusions:**

In conclusion, our findings highlight the impact of the COVID-19 pandemic on hospitalization patterns, equipping healthcare managers to improve resource allocation and readiness for future health challenges.

## Introduction

Coronavirus disease 2019 [1] pandemic has caused significant changes to healthcare systems throughout the world [2]. This virus was initially discovered in Wuhan, Hubei Province, China, in late 2019. From there, it quickly spread to other nations [3] and various strategies including social distancing and self-isolation were implemented by healthcare authorities to limit the impact of the spreading virus [4,5]. These regulations, along with the COVID-19 pandemic, seriously impacted peoples’ daily lives all around the world and had an immense impact on health care services. During pandemic, hospitals were responsible for diagnostic-therapeutic measures of COVID-19 patients beside providing routine services to other patients, which put more burden on them.

At the time, each country used different strategies to meet the challenges of personnel and bed shortage, providing diagnostic equipment and treatment medications [6]. Clinicians integrated virtual ways to visit their patients. Surgeons cancelled elective surgeries and in-person visits were limited to emergency cases. As health facilities adjusted to care for COVID-19 patients, the general public’s anxiety about visiting hospitals for other medical conditions increased [7,8]. Many patients even with life threatening medical conditions avoided seeking hospital care due to fear of infection [9] and in many countries the number of hospital admissions for other conditions was reduced [10,11]. In some countries, daily emergency visits were reduced but intensive care unit (ICU) admissions were increased [2]. In this regard, a survey in United Kingdom showed that parents avoid bringing their child to emergency department due to various reasons including sense of being infected at hospitals [12].

To the best of our knowledge, disease characteristics and outcomes are different among various populations and settings [13] as local population trends and socio-demographic characteristics influence the spread and impact of COVID-19. Evaluating the pattern of hospital admissions is crucial for understanding the broader impacts of the pandemic on healthcare systems [14]. In this context, we aimed to compare the admission rates of patients before and during the COVID-19 pandemic in a tertiary pediatric referral center in Iran, a country that faced a high prevalence of COVID-19 and later initiation of vaccination programs compared to developed nations [15,16]. By comparing admission patterns before and during the pandemic, this study seeks to provide insights that may help healthcare managers optimize resources and strategies in similar crises.

## Methods

### Study settings

In this retrospective study, we used data from health information system of 17 Shahrivar referral pediatric hospital center in Rasht city, the north of Iran. This is the only pediatric specialty hospital that caters exclusively to healthcare needs of children in Guilan region, Iran. This research was approved by the ethics committee at Guilan University of Medical Sciences (code: IR.GUMS.REC.1403.360), and the data for this research was accessed following the approval of data access permissions on October 24, 2024.

### Data source

The first case of COVID-19 diagnosed at February 2020. The data extracted for two periods, before COVID-19 pandemic (23 July 2017–23 January 2020(: and during the covid-19 pandemic (20 February 2020- 20 September 2022) of all hospitalized patients. We recorded variables including age, sex, duration of hospitalization, frequency of admission and outcome at discharge (remission or mortality). Disease categories recorded based on ICD-10 categories. The description of each ICD-10 code is as following: A00–B99: Certain infectious and parasitic diseases,C00–D48:Neoplasms, D50–D89: Diseases of the blood and blood-forming organs and certain disorders involving the immune mechanism, E00–E90: Endocrine, nutritional and metabolic diseases, F00–F99: Mental and behavioral disorders, G00–G99: Diseases of the nervous system, H00–H59: Diseases of the eye and adnexa, H60–H95: Diseases of the ear and mastoid process, I00–I99: Diseases of the circulatory system, J00–J99: Diseases of the respiratory system, K00–K93: Diseases of the digestive system, L00–L99: Diseases of the skin and subcutaneous tissue, M00–M99: Diseases of the musculoskeletal system and connective tissue, N00–N99: Diseases of the genitourinary system, O00–O99: Pregnancy, childbirth and the puerperium, P00–P96: Certain conditions originating in the perinatal period, Q00–Q99: Congenital malformations, deformations and chromosomal abnormalities, R00–R99: Symptoms, signs and abnormal clinical and laboratory findings, not elsewhere classified, S00–T98: Injury, poisoning and certain other consequences of external causes, V01–Y98: External causes of morbidity and mortality, Z00–Z99: Factors influencing health status and contact with health services, U00–U99: Codes for special purposes [17].

We also collected the number of hospitalized patients of each unit, including emergency, infectious, internal disorders, pediatric intensive care unit (PICU), neonatal intensive care unit (NICU), neonatal, hematology and surgery departments.

### Statistical analysis

Data were reported by number, percent, mean, and standard deviation. The normality of quantitative variables was assessed by the Kolmogorov Smirnov. The qualitative variables were compared before and during the COVID-19 by the chi-square test. Regarding the normal distribution of the quantitative variables, they were compared by the independent sample T-test. P-value<0.05 indicated statistical significance. Data analysis was performed by the IBM SPSS Statistics for Windows, Version 21.0. Armonk, NY: IBM Corp.

## Results

Characteristics of hospitalized children before and during COVID-19 pandemic are presented in Table 1. There was a significant difference between sex and age distribution before and during the pandemic (P=0.041, P<0.001). Comparing these two periods showed a decreasing trend in terms of the frequency of admission (14922 vs. 10941 patients). During the pandemic, patients experienced significantly shorter hospital stays (P<0.001). Also, there was no significant difference in the number of patients entering remission or those facing mortality (P=0.063).

**Table 1:** Comparing demographic and clinical characteristics of patients before and COVID-19 outbreak.

	Before COVID-19 outbreak	During COVID-19 outbreak	P-value
Age (mean ± SD)	3.56±0.036	3.87±0.041	<0.001
Sex			
Female	6245(43%)	4842(44.3%)	0.041
Male	8274(57%)	6089(55.7%)	
Duration of Hospitalization (mean ± SD, days)	4.36±0.046	3.96±0.046	<0.001
The frequency of readmission (mean ± SD)	1.98±0.030	1.76±0.049	<0.001
Outcome at discharge N (%)			
Remission	14798 (99.3%)	10846(99.1%)	0.063
Mortality	98 (0.7%)	94(0.9%)	

The number of patients with each ICD-10 diagnosis are presented in Table 2, indicating a statistically significant difference before and during COVID-19 (P<0.001). The results showed that neoplasms, disorders of blood, nervous system, eye, circulatory system, digestive system, genitourinary system, congenital malformation and poisoning had a significant rise during the pandemic while others had decreased number of admissions during the pandemic.

**Table 2:** Comparing diagnosis based on ICD-10 criteria in patients before and after COVID-19 outbreak.

ICD-10	Description	Before COVID-19 outbreak	During COVID-19 outbreak	p-value
A00–B99	Certain infectious and parasitic diseases	3394 (22.7%)	2221 (20.3%)	<0.001
C00–D48	Neoplasms	327 (2.2%)	249 (2.3%)	
D50–D89	Diseases of the blood and blood-forming organs and certain disorders involving the immune mechanism	927 (6.2%)	847 (7.7%)	
E00–E90	Endocrine, nutritional and metabolic diseases	822 (5.5%)	588 (5.4%)	
F00–F99	Mental and behavioral disorders	268 (1.8%)	112 (1.0%)	
G00–G99	Diseases of the nervous system	605 (4.1%)	555 (5.1%)	
H00–H59	Diseases of the eye and adnexa	72 (.5%)	65 (.6%)	
H60–H95	Diseases of the ear and mastoid process	96 (.6%)	16 (.1%)	
I00–I99	Diseases of the circulatory system	192 (1.3%)	152 (1.4%)	
J00–J99	Diseases of the respiratory system	1798 (12.0%)	654 (6.0%)	
K00–K93	Diseases of the digestive system	1092 (7.3%)	846 (7.7%)	
L00–L99	Diseases of the skin and subcutaneous tissue	224 (1.5%)	134 (1.2%)	
M00–M99	Diseases of the musculoskeletal system and connective tissue	214 (1.4%)	115 (1.1%)	
N00–N99	Diseases of the genitourinary system	467 (3.1%)	350 (3.2%)	
O00–O99	Pregnancy, childbirth and the puerperium	734 (4.9%)	658 (6.0%)	
P00–P96	Certain conditions originating in the perinatal period	10 (.1%)	5 (.0%)	
Q00–Q99	Congenital malformations, deformations and chromosomal abnormalities	1657 (11.1%)	1552 (14.2%)	
R00–R99	Symptoms, signs and abnormal clinical and laboratory findings, not elsewhere classified	282 (1.9%)	132 (1.2%)	
S00–T98	Injury, poisoning and certain other consequences of external causes	1713 (11.5%)	1409 (12.9%)	
V01–Y98	External causes of morbidity and mortality	15 (.1%)	0 (.0%)	
Z00–Z99	Factors influencing health status and contact with health services	9 (.1%)	26 (.2%)	
U00–U99	Codes for special purposes	0 (.0%)	90 (.8%)	
Total		14922 (100.0%)	10941 (100.0%)	

According to the results of Table 3, the number of admissions in each ward was significantly different before and during the pandemic and we noticed a significant rise in admissions of NICU, PICU, emergency, neonatal, and hematology departments during the pandemic (P<0.001).

**Table 3:** Comparing admission rate of each ward before and during COVID-19 outbreak.

			Before COVID-19 outbreak	During COVID-19 outbreak	p-value
Ward	NICU	Number	257	267	<0.001
		%	1.7%	2.4%	
	PICU	Number	187	250	
		%	1.3%	2.3%	
	Emergency	Number	7171	5590	
		%	48.1%	51.1%	
	Surgery	Number	369	235	
		%	2.5%	2.1%	
	Infectious diseases	Number	1415	714	
		%	9.5%	6.5%	
	Neonatal	Number	1888	1614	
		%	12.7%	14.8%	
	Hematology	Number	614	505	
		%	4.1%	4.6%	
	Internal disorders 1	Number	1920	974	
		%	12.9%	8.9%	
	Internal disorders 2	Number	1101	792	
		%	7.4%	7.2%	
Total		Number	14922	10941	
		%	100.0%	100.0%	

*PICU: Pediatric Intensive Care Unit, NICU: Neonatal Intensive Care Unit.

## Discussion

The COVID-19 pandemic has changed the healthcare utilizations, worldwide, with profound impact on non-COVID-related diseases [17,18]. Therefore, it is crucial to study these changes and find out the pattern of change for a better management of health systems in case of any future pandemics. Thus, in this study, we investigated the hospitalization rates of all ICD-10 disease categorizations at the 17-Shahrivar hospital, which is the only tertiary referral pediatric hospital in Guilan Province of Iran.

The results of this research showed a significant reduction in the number of hospitalizations during the COVID-19 pandemic compared to the same periods in the two previous years which is in line with the result of a study conducted by Ahmadi et al. [19]. In line with our results, in China, the overall hospitalizations decreased by 26.35% during the pandemic in both adults and pediatrics [20,21]. Also, In Turkey, pediatric admissions decreased by 63.11%, with significant reductions in respiratory, gastrointestinal, and immunological diseases, while neonatal, urogenital, and cardiovascular cases increased during the COVID-19 lockdown [22]. Decreased admissions during the pandemic may be due to several reasons including the fear of contracting the infection and preferring to get at-home treatments for mild-severity diseases [19]. A survey in Canada demonstrated that the greatest decline was observed in preventive visits for patients with chronic diseases [23], but there is also some evidence in Germany and California showing that patients with life-threatening disorders like ischemic cerebrovascular events and acute myocardial infarction have also avoided hospital visits [24,25].

There was an increasing trend in terms of categories including neoplasms, diseases of blood, nervous system, eye and adnexa, circulatory, genitourinary and digestive systems, congenital malformations, poisonings and diseases with uncertain etiologies. Other categories including infectious diseases and respiratory disorders had lower hospital admissions during pandemic rather than before. In this regard, a dramatic decrease in all non-SARS-CoV-2 respiratory pathogens was seen globally in the beginning of the pandemic [26]. In case of investigating the prevalence of cases affected by other respiratory viruses, a survey in England showed a significant reduction in patients with respiratory syncytial virus among children under 5 years old during the pandemic compared to pre-pandemic time [27]. Reduction of respiratory syncytial virus was also reported in Australia, United States, and Italy during the pandemic [28–30]. Moreover, positive cases of Hemophilus influenza infections in children had dropped by 6.21% in 2020 and 7.37% in 2021 [31] and the incidence of bacterial infections in children had a dramatic decline in early 2020 in 26 countries [32]. The probable explanation for this change might be the suppression of Co-pathogens that could cause secondary bacterial infections, including RSV, influenza viruses, and human metapneumovirus [33].

Our results demonstrated a significant reduction in the hospitalization rate of patients with endocrine disorders. The relationship between COVID-19 and endocrine disorders has been investigated in some previous studies. Angiotensin-converting enzyme 2 is a membrane enzyme that SARS-COV-2 binds to in order to interact with host cells. Since Angiotensin-converting enzyme 2 is expressed in many glands that have endocrine functions, this interaction can directly affect endocrine function in patients with COVID-19 infection. In addition, many endocrine disorders can change the susceptibility of contracting COVID-19 infection. For example, in obesity due to the higher chronic inflammatory status and imbalance metabolic regulations the risk of COVID infection increases [34]. Also, patients with diabetes mellitus have higher susceptibility of contracting COVID-19 [35,36], but physicians educated patients to monitor their blood glucose by themselves and get teleconsultation as well as educations about sick-day guidelines [36,37]. In this regard, the observed decrease in hospitalized patients with endocrine disorders during the pandemic in our results can be contributed to increased use of telemedicine and self-monitoring.

Our findings also indicated that hospitalizations due to mental disorders had fell during the pandemic while a study of 8 pediatric hospitals in the United States and France showed that patients with mental disorders had a higher monthly proportion of hospitalizations with the onset of pandemic [1]. Other diseases with a decreasing trend with the start of pandemic are diseases of ear, skin, musculoskeletal system, childbirth and perinatal diseases. They are presented as less urgent conditions compared to a range of other medical emergencies, leading to lower hospitalization rates especially during the pandemic that patients preferred virtual consultations rather than in-person visits.

After assessing all the wards, the number of hospitalization rates showed that surgery, infectious diseases, and internal disorders wards had a significant decrease. The decreased rate of surgery ward’s admission can be due to recommendations for postponing the elective surgeries to dedicate enough health source for patients in need of urgent care [38].

Furthermore, a survey in Portugal showed a significant increase in in-hospital mortality of patients hospitalized in internal wards with the start of COVID-19 pandemic [39]. The increased mortality rate may be because patients with internal diseases attended hospital when they were in severe conditions and they missed on-time treatments. In our results, we did not observe a significant difference in the hospital’s total mortality rate, but the decreased rate of hospitalizations in our internal diseases ward indicate that patients preferred to get at-home treatments during the pandemic.

## Conclusion

In conclusion, the COVID-19 pandemic has profoundly changed the hospitalization patterns. Our results demonstrated the rates of admission and mortality associated with various diseases, categorized using ICD-10 codes. This information is crucial for healthcare managers as it enables them to identify unnecessary hospital admissions and pinpoint patients who may experience complications and higher mortality rates. With this knowledge, managers can allocate healthcare resources more effectively and better prepare for future public health crises.

## References

[1] Gutiérrez-Sacristán A, Serret-Larmande A, Hutch M, Sáez C, Aronow B, Bhatnagar S. Hospitalizations associated with mental health conditions among adolescents in the US and France during the COVID-19 pandemic. JAMA Network Open. 2022;5(12):e2246548. doi: 10.1001/jamanetworkopen.2022.46548 36512353 PMC9856226

[2] Rennert-May E, Leal J, Thanh NX, Lang E, Dowling S, Manns B, et al. The impact of COVID-19 on hospital admissions and emergency department visits: a population-based study. PLoS One. 2021;16(6):e0252441. doi: 10.1371/journal.pone.0252441 34061888 PMC8168854

[3] Roknuzzaman A, Sarker R, , Shahriar M, Mosharrafa RA, Islam MR. The WHO has declared COVID-19 is no longer a pandemic-level threat: a perspective evaluating potential public health impacts. Clin Pathol. 2024;17:2632010X241228053. doi: 10.1177/2632010X241228053 38264675 PMC10804921

[4] Nicola M, O’Neill N, Sohrabi C, Khan M, Agha M, Agha R. Evidence based management guideline for the COVID-19 pandemic - review article. Int J Surg. 2020;77:206–16. doi: 10.1016/j.ijsu.2020.04.001 32289472 PMC7151371

[5] Hu C-Y, Tang Y-W, Su Q-M, Lei Y, Cui W-S, Zhang Y-Y, et al. Public health measures during the COVID-19 pandemic reduce the spread of other respiratory infectious diseases. Front Public Health. 2021;9:771638. doi: 10.3389/fpubh.2021.771638 34858936 PMC8631357

[6] Mohammadinia L, Saadatmand V, Khaledi Sardashti H, Darabi S, Esfandiary Bayat F, Rejeh N, et al. Hospital response challenges and strategies during COVID-19 pandemic: a qualitative study. Front Public Health. 2023;11:1167411. doi: 10.3389/fpubh.2023.1167411 37457272 PMC10349376

[7] Lazzerini M, Barbi E, Apicella A, Marchetti F, Cardinale F, Trobia G. Delayed access or provision of care in Italy resulting from fear of COVID-19. Lancet Child Adolesc Health. 2020;4(5):e10–1. doi: 10.1016/S2352-4642(20)30108-5 32278365 PMC7146704

[8] Hung KK, Walline JH, Chan EYY, Huang Z, Lo ESK, Yeoh EK, et al. Health service utilization in hong kong during the COVID-19 pandemic - a cross-sectional public survey. Int J Health Policy Manag. 2022;11(4):508–13. doi: 10.34172/ijhpm.2020.183 33105965 PMC9309937

[9] Birkmeyer JD, Barnato A, Birkmeyer N, Bessler R, Skinner J. The impact of the COVID-19 pandemic on hospital admissions in the United States. Health Aff (Millwood). 2020;39(11):2010-7. 32970495 10.1377/hlthaff.2020.00980 PMC7769002

[10] Markus HS, Brainin M. COVID-19 and stroke-a global world stroke organization perspective. Int J Stroke. 2020;15(4):361–4. doi: 10.1177/1747493020923472 32310017 PMC11927026

[11] Metzler B, Siostrzonek P, Binder RK, Bauer A, Reinstadler SJ. Decline of acute coronary syndrome admissions in Austria since the outbreak of COVID-19: the pandemic response causes cardiac collateral damage. Eur Heart J. 2020;41(19):1852–3. doi: 10.1093/eurheartj/ehaa314 32297932 PMC7184486

[12] Breckons M, Thorne S, Walsh R, Bhopal S, Owens S, Rankin J. Parental perspectives on emergency health service use during the first wave of the COVID-19 pandemic in the United Kingdom: a qualitative study. PLoS One. 2023;18(5):e0285375. doi: 10.1371/journal.pone.0285375 37256845 PMC10231793

[13] Edae G, Tekleab AM, Getachew M, Bacha T. Admission pattern and treatment outcome in pediatric intensive care unit, Tertiary Hospital, Addis Ababa, Ethiopia. Ethiop J Health Sci. 2022;32(3):497–504. doi: 10.4314/ejhs.v32i3.4 35813669 PMC9214737

[14] Damiri S, Shojaee A, Dehghani M, Shahali Z, Abbasi S, Daroudi R. National geographical pattern of COVID-19 hospitalization, case fatalities, and associated factors in patients covered by Iran Health Insurance Organization. BMC Public Health. 2022;22(1):1274. doi: 10.1186/s12889-022-13649-0 35773657 PMC9243909

[15] Khosravi A, Chaman R, Rohani-Rasaf M, Zare F, Mehravaran S, Emamian M. The basic reproduction number and prediction of the epidemic size of the novel coronavirus (COVID-19) in Shahroud, Iran. Epidemiol Infect. 2020;148:e115. 32517845 10.1017/S0950268820001247 PMC7322167

[16] Heidari M, Jafari H. Challenges of COVID-19 vaccination in Iran: in the fourth wave of pandemic spread. Prehosp Disaster Med. 2021;36(5):659–60. doi: 10.1017/S1049023X21000777 34287113 PMC8365040

[17] Rezaei Z, Lotfi F, Bayati M, Kavosi Z. The effect of Covid-19 pandemic on healthcare utilization in public vs private centers in Iran: a multiple group interrupted time-series analysis. BMC Health Serv Res. 2023;23(1):822. doi: 10.1186/s12913-023-09846-1 37528374 PMC10394764

[18] Mrożek-Gąsiorowska M, Tambor M. How COVID-19 has changed the utilization of different health care services in Poland. BMC Health Serv Res. 2024;24(1):105. doi: 10.1186/s12913-024-10554-7 38238694 PMC10797947

[19] Ahmadi S, Kazemi-Karyani A, Badiee N, Byford S, Mohammadi A, Piroozi B, et al. The impact of COVID-19 pandemic on hospital admissions for nine diseases in Iran: insight from an interrupted time series analysis. Cost Eff Resour Alloc. 2022;20(1):58. doi: 10.1186/s12962-022-00394-9 36319966 PMC9628075

[20] Yang Y, Le K-J, Liang C, Zheng T, Gu Z-C, Lin H-W, et al. Changes in inpatient admissions before and during COVID-19 outbreak in a large tertiary hospital in Shanghai. Ann Transl Med. 2022;10(8):469. doi: 10.21037/atm-22-1594 35571407 PMC9096364

[21] Yan H, Li X, Lu X, Zeng S, Yuan Y, Hu X, et al. Changes in pediatric healthcare utilization in Hunan Province, China, during the COVID-19 pandemic: a multi-center cross-sectional study. Transl Pediatr. 2021;10(4):870–81. doi: 10.21037/tp-20-465 34012836 PMC8107880

[22] Erdede O, Sarı E, Uygur Külcü N, Sezer Yamanel R. The impact of COVID-19 lockdown on pediatric hospital admissions in Turkey. J Pediatr Infect Dis. 2022;17(05):227–33.

[23] Stephenson E, Butt DA, Gronsbell J, Ji C, O’Neill B, Crampton N, et al. Changes in the top 25 reasons for primary care visits during the COVID-19 pandemic in a high-COVID region of Canada. PLoS One. 2021;16(8):e0255992. doi: 10.1371/journal.pone.0255992 34383844 PMC8360367

[24] Hoyer C, Ebert A, Huttner HB, Puetz V, Kallmünzer B, Barlinn K, et al. Acute stroke in times of the COVID-19 pandemic: a multicenter study. Stroke. 2020;51(7):2224–7. doi: 10.1161/STROKEAHA.120.030395 32516064

[25] Solomon M, McNulty E, Rana J, Leong T, Lee C, Sung S. The Covid-19 pandemic and the incidence of acute myocardial infarction. N Engl J Med. 2020;383(7):691–3. 32427432 10.1056/NEJMc2015630

[26] Principi N, Autore G, Ramundo G, Esposito S. Epidemiology of respiratory infections during the COVID-19 pandemic. Viruses. 2023;15(5). 10.3390/v15051160 PMC10224029 37243246

[27] Torres AR, Guiomar RG, Verdasca N, Melo A, Rodrigues AP, Rede Portuguesa de Laboratórios para o Diagnóstico da Gripe. Resurgence of respiratory syncytial virus in children: an out-of-season epidemic in Portugal. Acta Med Port. 2023;36(5):343–52. doi: 10.20344/amp.18589 36705636

[28] Nenna R, Matera L, Licari A, Manti S, Di Bella G, Pierangeli A. An Italian multicenter study on the epidemiology of respiratory syncytial virus during SARS-CoV-2 pandemic in hospitalized children. Front Pediatr. 2022;10:930281. 35911833 10.3389/fped.2022.930281 PMC9329524

[29] Britton PN, Hu N, Saravanos G, Shrapnel J, Davis J, Snelling T, et al. COVID-19 public health measures and respiratory syncytial virus. Lancet Child Adolesc Health. 2020;4(11):e42–3. doi: 10.1016/S2352-4642(20)30307-2 32956616 PMC7500894

[30] Olsen S, Winn A, Budd A, Prill M, Steel J, Midgley C, et al. Changes in influenza and other respiratory virus activity during the COVID-19 pandemic - United States, 2020-2021. MMWR Morb Mortal Wkly Rep. 2021;70(29):1013–9. 34292924 10.15585/mmwr.mm7029a1 PMC8297694

[31] Zhou J, Zhao P, Nie M, Gao K, Yang J, Sun J. Changes of Haemophilus influenzae infection in children before and after the COVID-19 pandemic, Henan, China. J Infect. 2023;86(1):66–117. doi: 10.1016/j.jinf.2022.10.019 36273637 PMC9614014

[32] Brueggemann AB, Jansen van Rensburg MJ, Shaw D, McCarthy ND, Jolley KA, Maiden MCJ, et al. Changes in the incidence of invasive disease due to Streptococcus pneumoniae, Haemophilus influenzae, and Neisseria meningitidis during the COVID-19 pandemic in 26 countries and territories in the Invasive Respiratory Infection Surveillance Initiative: a prospective analysis of surveillance data. Lancet Digit Health. 2021;3(6):e360–70. doi: 10.1016/S2589-7500(21)00077-7 34045002 PMC8166576

[33] Willen L, Ekinci E, Cuypers L, Theeten H, Desmet S. Infant pneumococcal carriage in belgium not affected by COVID-19 containment measures. Front Cell Infect Microbiol. 2022;11:825427. doi: 10.3389/fcimb.2021.825427 35111700 PMC8801737

[34] Gnocchi M, D’Alvano T, Lattanzi C, Messina G, Petraroli M, Patianna VD, et al. Current evidence on the impact of the COVID-19 pandemic on paediatric endocrine conditions. Front Endocrinol (Lausanne). 2022;13:913334. doi: 10.3389/fendo.2022.913334 35992140 PMC9388786

[35] Pal R, Bhansali A. COVID-19, diabetes mellitus and ACE2: The conundrum. Diabetes Res Clin Pract. 2020;162:108132. doi: 10.1016/j.diabres.2020.108132 32234504 PMC7118535

[36] Banerjee M, Chakraborty S, Pal R. Diabetes self-management amid COVID-19 pandemic. Diabetes Metab Syndr. 2020;14(4):351–4. doi: 10.1016/j.dsx.2020.04.013 32311652 PMC7194953

[37] Ghosh A, Gupta R, Misra A. Telemedicine for diabetes care in India during COVID19 pandemic and national lockdown period: guidelines for physicians. Diabetes Metab Syndr. 2020;14(4):273–6. doi: 10.1016/j.dsx.2020.04.001 32283497 PMC7129346

[38] Mehta A, Awuah WA, Ng JC, Kundu M, Yarlagadda R, Sen M, et al. Elective surgeries during and after the COVID-19 pandemic: case burden and physician shortage concerns. Ann Med Surg (Lond). 2022;81:104395. doi: 10.1016/j.amsu.2022.104395 35999832 PMC9388274

[39] Ramalho AR, Mendes AC, Camões G, Roque R, Moura P, Mateus-Pinheiro A, et al. Impact of COVID-19 pandemic on in-hospital mortality in patients without SARS-CoV-2 infection in an internal medicine ward of a Tertiary Care Hospital in Portugal. Cureus. 2022;14(11):e32059. doi: 10.7759/cureus.32059 36600838 PMC9802641

